# Amplitude- and Phase-Programmable Dual-Color Photonic Chip for High-Contrast Structured Illumination Microscopy

**DOI:** 10.1021/acsphotonics.5c02733

**Published:** 2026-02-09

**Authors:** Paolo Maran, Abhiram Rajan, Francesco Ceccarelli, Roberto Osellame, Petra Paiè, Alessia Candeo, Francesca Bragheri, Andrea Bassi

**Affiliations:** † Dipartimento di Fisica, 18981 Politecnico di Milano, Milan 20133, Italy; ‡ Istituto di Fotonica e Nanotecnologie (IFN), Consiglio Nazionale delle Ricerche (CNR), Milan 20133, Italy

**Keywords:** structured illumination microscopy, HiLo microscopy, integrated optics, femtosecond laser micromachining

## Abstract

Advanced optical microscopy techniques, such as structured illumination microscopy (SIM), often rely on precise and complex illumination setups, which can be challenging to implement and maintain. Integrated optics can offer compact, stable, and easy-to-align alternatives, enabling efficient light manipulation for advanced imaging applications. We present an integrated photonic device that generates structured illumination patterns directly within an optical microscope. The device incorporates optical waveguides in a Mach–Zehnder interferometer configuration, generating two separate coherent point sources with controlled amplitudes and phases. When optically conjugated to the pupil plane of a conventional widefield microscope, the device generates sinusoidal illumination patterns in the object plane, which can be translated and modulated via the Mach–Zehnder interferometer. We demonstrate that amplitude modulation enables (i) optical sectioning in HiLo (High and Low Frequency Illumination) microscopy and (ii) controlled structured illumination contrast across multiple wavelengths, making the system adaptable for multicolor SIM. Our results highlight the potential of integrated photonics as a compact and robust approach for advanced microscopy techniques, contributing to the development of simplified, high-resolution structured illumination imaging in biomedical and materials science applications.

## Graphical abstract

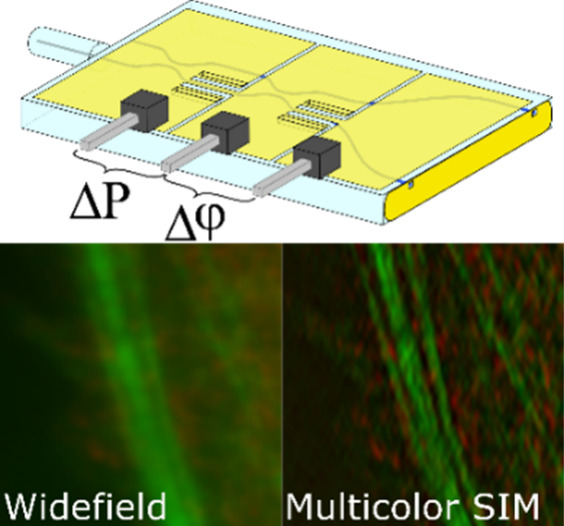

## Introduction

Optical microscopy is a fundamental tool in both material science and biomedicine. It enables the detailed analysis of microstructures in engineered materials and allows visualization of cells and tissues at subcellular resolution in biological systems. However, many advanced microscopy techniques depend on complex illumination schemes that require precise optical alignment, which can hinder their integration into standard laboratory setups. Among these, structured illumination microscopy (SIM) has emerged as a particularly powerful approach for enhancing image contrast, enabling optical sectioning,^1,2^ and surpassing the diffraction limit in fluorescence imaging.^3,4^ SIM works by projecting a series of sinusoidal light patterns onto the sample and acquiring multiple phase-shifted images, which are then computationally combined to reconstruct fine spatial details. Traditionally, generating these structured patterns has required free-space optical components, such as diffraction gratings or spatial light modulators, positioned in the excitation path. These elements introduce alignment challenges, mechanical instabilities, and contribute to the overall complexity of the system.

Significant research and development efforts have been dedicated to building compact SIM systems, with the aim of increasing stability and reducing the system complexity and size, in order to enable the use of SIM in point-of-care applications.^5–8^ Toward this goal, integrated photonic solutions provide a compelling alternative, enabling compact, robust, and precisely controlled light modulation for microscopy applications. Several research efforts have explored the potential of fiber based or integrated photonic devices for structured illumination microscopy.^9–13^ These approaches leverage the advantages of photonic waveguides and interferometric structures to miniaturize and stabilize illumination control. Previously, we developed an integrated photonic chip capable of generating structured illumination for far-field SIM, employing a phase shifter and three optical fibers to create a hexagonal illumination pattern.^14^ This demonstrated the feasibility of integrated structured illumination.

While these recent advances have made SIM more compact and accessible, existing implementations compromise on one or more features: lack of amplitude control in integrated/fiber devices, limited spectral operation (single-wavelength use), or reliance on complex bulk optics other than simple lenses and mirrors. Furthermore, fiber technology-based solutions can suffer from stability issues due to mechanical vibrations of the fibers themselves. Here, we present an integrated photonic SIM illuminator that overcomes these limitations. Our device is built around an on-chip Mach–Zehnder interferometer (MZI) that acts as a tunable beam splitter for amplitude modulation, paired with an independent thermo-optic phase shifter for precise fringe position tuning. In addition, a custom-fabricated spatial filter mask is introduced for background suppression, ensuring >90% fringe contrast on the sample. This architecture enables us to generate high-visibility sinusoidal illumination, a key requirement for SIM microscopy.

When coupled to an external commercial widefield microscope, the photonic-chip platform provides a common-path optical scheme that is intrinsically phase-stable, eliminating phase drift between arms and maintaining fringe phase stability over long acquisitions. Notably, our system supports multicolor SIM operation by design: the waveguide interferometer and mask can be used across multiple excitation wavelengths, which we demonstrate with dual-color structured illumination. Furthermore, the controllable MZI allows us to switch between structured and uniform illumination, enabling High and Low Frequency Illumination (HiLo) optical sectioning.^2^ To our knowledge, this is the first integrated photonic SIM device offering simultaneous amplitude and phase programmability with multiwavelength capability. In contrast to previous integrated or fiber-based SIM^9–13^ implementations, the proposed device combines independent and programmable amplitude and phase control within a single monolithic photonic device, removing pattern instabilities caused by mechanical vibrations of optical fibers while enabling multicolor operation. This integration enables high-contrast structured illumination, controlled phase stepping, and switching between uniform and patterned illumination without moving parts. This technology can also be effectively used in fully integrated SIM systems^11–13^ to enable multicolor illumination or contrast modulation.

In the following, we detail the design and fabrication of the device. We then evaluate contrast, phase stability and intensity balance across multiple wavelengths. Our results position this integrated photonic approach as a significant advancement in SIM instrumentation, combining the compact robustness of on-chip optics with the full control and versatility required for state-of-the-art super-resolution microscopy. We demonstrate the capabilities of the integrated device in HiLo microscopy^2^ and multicolor SIM,^3^ showing how the chip can be adopted for optical sectioning and resolution enhancement. The ability to dynamically modulate both amplitude and phase further extends its versatility, making it suitable for a wide range of photonic and imaging applications beyond microscopy, including optical trapping, interferometry, and profilometry.

## Materials and Methods

### Chip-Based Optical Microscope Scheme

The structured illumination patterns used in this work are generated by an integrated photonic device (Figure 1) specifically designed for direct coupling into a conventional widefield microscope. The device combines multiple optical functions, including beam splitting, amplitude tuning, and phase modulation, onto a single glass substrate. This enables the generation of two point sources, controlled in amplitude and phase, which produce by interference a sinusoidal pattern in the far field. Amplitude and phase difference control is achieved without mechanical movements by exploiting the thermo-optical effect via electrical resistors fabricated on top of the chip’s surface. The pattern generator is coupled to an inverted microscope (Nikon Eclipse Ti2U) using a lens system (Figure 1c), in order to generate the interference pattern at the image plane of the microscope side port which is optically conjugated to the sample plane. The chip outputs are imaged using a 4f system, comprised of objective lens L1 (Leica N PLAN 5×/0.12) and lens L2 (*f*_2_ = 150 mm) in the figure onto the focal plane of lens L3 (*f*_3_ = 100 mm), which collimates the beams and generates the interference pattern at the desired plane. The microscope tube lens and objective lens then image this pattern on the sample plane, generating the structured illumination. The pattern period on the sample plane, which defines the achievable resolution enhancement in SIM, depends on the separation between the two beams at the objective lens pupil plane. This distance can be tuned by changing the physical distance of the chip outputs or by changing the optical magnification between chip output facet and pupil plane, which is equal to the ratio of focal lengths f2fTf1f3 , (Figure 1c) where *f*_T_ is the focal length of the microscope tube lens. However, higher magnifications will result in smaller fields of illumination; therefore, we chose to keep the magnification between the chip facet and the pupil plane low. The fluorescent emission excited in the sample is collected by the objective lens and propagated up to the side port of the microscope, where a multiband dichroic mirror (Semrock FL-007018) and a detection filter (Semrock FL-004586) separate the illumination and detection paths. Afterward, a relay system with unitary magnification is used to image the sample onto the sensor of a CMOS camera (Hamamatsu ORCA-Fusion CP15440-20UP).

**Figure 1:**
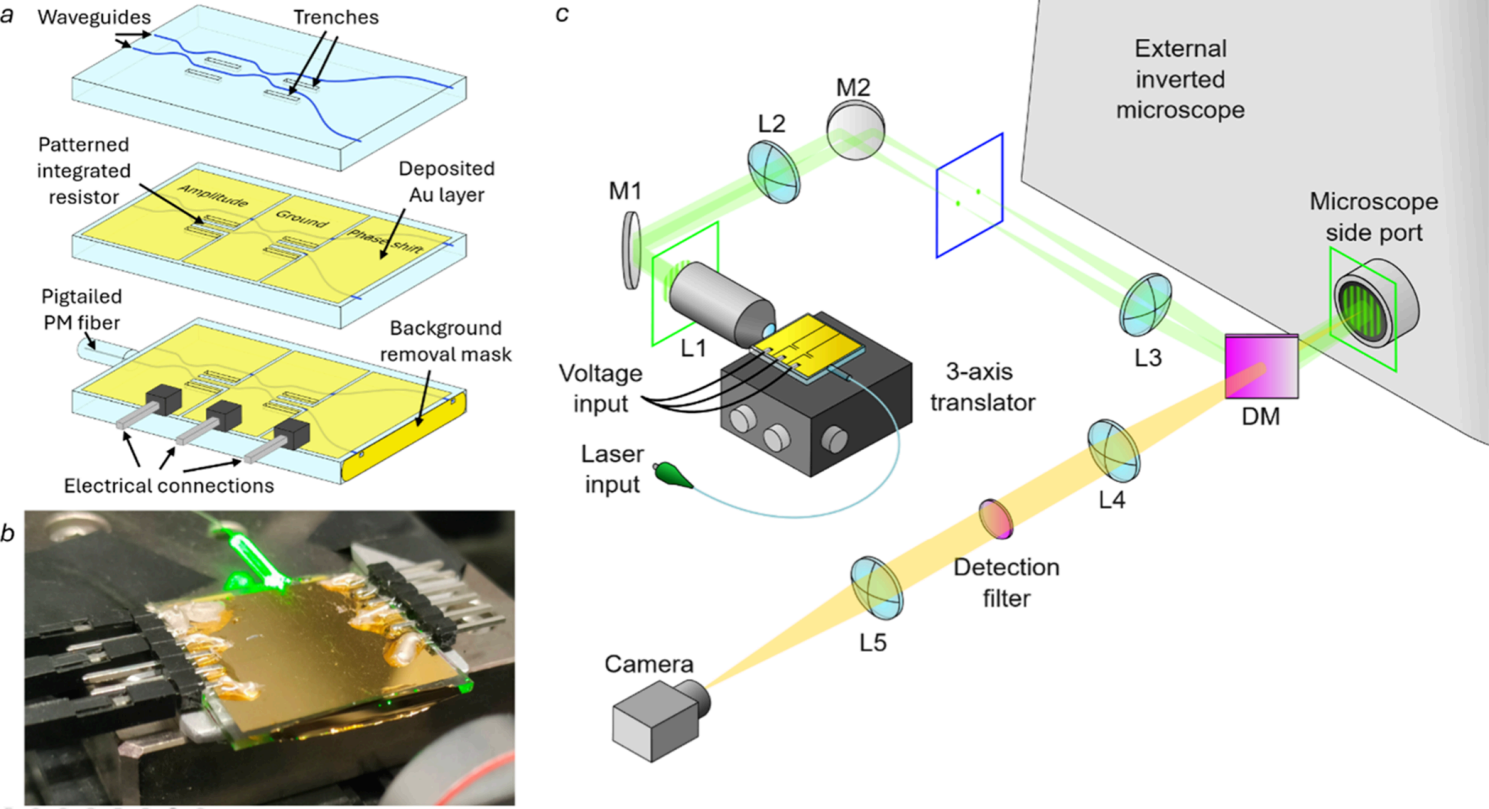
Chip layout and optical system schematics. (a) Integrated pattern generator layout at different fabrication stages showing waveguides and trenches (top), deposited gold for resistive thermal phase shifter patterning (center), and connectors and mask for background removal (bottom). (b) Close-up picture of the pattern generator. (c) Schematic view of the optical system used to generate structured illumination on an external inverted microscope (L for lenses, M for mirrors, and DM for dichroic mirror).

In the SIM implementation described below, the used microscope objective (Nikon CFI Apochromat TIRF 60XC Oil) had a numerical aperture of NA = 1.49 and a magnification of M = 60×, the pupil diameter was *D* = 2NA *f*_Obj_ = 2NA *f*_T_/M = 9.9 mm. The pattern generator’s outputs separation was equal to *d*_chip_ = 1 mm. Given the optical magnification between the chip’s surface and the objective’s back focal plane, which is equal to f2fTf1f3 , the beams separation at the pupil plane was equal to dpp=f2fTf1f3dchip=7.5mm . In this way, a theoretical resolution enhancement of F=1+dPPD=1.75 can be achieved.

It is worth noting that the use of relay optics to conjugate the pattern generator to the microscope pupil plane is inherent to far-field SIM implementation, including systems based on spatial light modulators, digital micromirror devices, fiber bundles, or diffractive optics. In this context, the integrated photonic chip replaces the most alignment-sensitive and mechanically unstable elements of the illumination path, while the remaining optics are passive, fixed, and compatible with compact microscope add-on architectures.

### Waveguides Fabrication

To implement the integrated photonic functions required for SIM such as waveguides, beam splitters, and phase modulators, the custom optical components must be embedded directly into a substrate. This necessitates a fabrication method capable of producing structures with micrometer-scale precision, minimal surface roughness, and low optical loss. Femtosecond laser micromachining (FLM) offers these capabilities in a versatile platform, enabling direct-writing fabrication of optical waveguides and interferometric circuits in glass substrates.^15^ FLM was used to fabricate waveguides and dig trenches directly onto a 25 × 25 mm^2^, 1 mm thick borosilicate substrate (Corning Eagle XG). This process works by exploiting nonlinear absorption phenomena which occur in the bulk of a transparent and dielectric material when a focused femtosecond laser is shined through it. When the beam is focused inside the substrate, the laser intensity exceeds the nonlinear absorption threshold only in a confined region surrounding the laser’s focal point; therefore, material modification can be induced in a small, controllable zone.

The nature of the material modification depends on the laser fluence deposited on the sample, which in turn can be controlled by tuning multiple experimental parameters such as average laser power, repetition rate, pulse width, scan speed, and numerical aperture of the focusing objective. In order to use this process to realize light-guiding devices, we must induce a smooth refractive index modification in the substrate, which can then be used to fabricate waveguides by translating the sample with respect to the laser’s focal spot. Furthermore, the fabricated waveguides must show single mode operation and low propagation losses at all wavelengths of interest; therefore, a critical part in the device’s fabrication is finding the correct waveguide fabrication parameters.

The optimal recipe was determined experimentally by exploring extensively the multidimensional fabrication parameter space. Using a 1030 nm wavelength, 190 fs pulse-width laser with 1 MHz repetition rate (Light Conversion CARBIDE CB5), multiple waveguides were fabricated by tuning the average laser power, the sample translation speed, and the number of repeated scans of the same waveguide. After identifying the most promising waveguides in each fabrication run, a narrower parameter range was explored to refine and determine the best possible parameter set. Through this iterative process, single mode waveguides with propagation losses as low as 0.3 dB/cm were achieved for both 638 and 561 nm illumination; the corresponding fabrication parameters were 250 mW average laser power, 45 mm/s sample translation speed and 2 scans for each waveguide. Waveguides were fabricated at a depth of 15 μm from the chip’s bottom surface. The fabricated waveguides exhibited slightly elliptical single-mode profiles, with 1/e^2^ mode diameters of 4.2 μm (horizontal) × 4.7 μm (vertical) at 638 nm, and 3.4 μm (horizontal) × 3.6 μm (vertical) at 561 nm.

By increasing the fluence deposited into the substrate, laser ablation can occur, resulting in material removal when the laser is focused near the chip’s surface. This fabrication regime can be exploited to fabricate groove-like trenches between adjacent waveguides. These structures are used to provide the best compromise between power dissipation and temporal response in the thermo-optic phase shifting process, as thoroughly described in ref 16.

Trenches measuring 3 mm in length, 10 μm in depth and 8 μm in width were fabricated by ablating three parallel lines spaced horizontally by 1 μm, using 310 mW average laser power and 4 mm/s translation speed, with two scans per line. This structure was repeated vertically, with a 2 μm vertical spacing, up to the desired trench depth. After trench fabrication, a short etching procedure (8 min into a 10 M KOH solution) was employed to remove any glass residue inside the trenches.

After FLM waveguide fabrication, a thermal annealing process is used to mitigate the internal stresses built up into the substrate during fabrication, which has been shown to improve the polarization insensitivity of the resulting waveguides.^17^ Afterward, 700 μm are removed from the chip’s input and output facets to expose the waveguides. After lapping, the chip’s input and output facets need to be polished to avoid losses in input coupling and mode distortions in output coupling due to the chip’s surface roughness. This process is performed via mechanical polishing (Logitech LP50) using alumina oxide powder with varying degrees of particle size (30–1 μm) as abrasive slurry to progressively reduce the surface roughness and a last polishing step with colloidal silica (30 nm) to provide the final chip surfaces with optimal quality.

### Mach–Zehnder Interferometers and Thermal Shifters Manufacturing

To enable amplitude control of the interference pattern, an MZI with a thermal phase shifter in one arm was integrated into the photonic chip (Figure 1a). By adjusting the relative optical power between the two output waveguides through a fine-tuning of the dissipated heat from the thermal phase shifter, the MZI allows the control of the interference contrast. A second thermal phase shifter fabricated on one of the two arms outside of the MZI modulates the phase between the outputs, enabling lateral translation of the structured illumination pattern. These optical functions are fully embedded in the glass substrate using FLM.

MZIs consist of two beam splitters connected by two optical paths, one of which includes a phase-shifting element. In the integrated version, the beam splitters are implemented as directional couplers (DCs) with coupling distance of ∼5.4 μm and zero coupling length, designed for a waveguide separation of 30 μm and for obtaining a 50% splitting ratio (SR), here defined as ratio between the output power in the interferometer arm corresponding to the one where light was input and the total output power of the device, at 638 nm. The waveguide depth was set equal to 15 μm. The waveguides before and after the couplers are curved, with a curvature radius equal to 40 mm to minimize bending losses, to achieve the desired value of the output separation, allowing interfacing with external optics and the background-suppression mask. Since this distance, together with the overall system magnification, determines the spatial frequency of the pattern generated at the sample plane, devices with different output distances can be used to obtain different patterns with varying spatial frequency based on the specific measurement requirements. We used an output separation of 500 μm for the measurements with the HiLo microscope (see Results and Discussion), corresponding to a pattern with period equal to 618 nm with an illumination wavelength of 561 nm, while the SIM images were acquired using a device with a 1 mm output distance, which generated a pattern at sample plane with 276 nm period for red illumination (λ = 638 nm) and 244 nm for green illumination (λ = 561 nm).

Thermal phase shifting is implemented by patterning gold resistive heaters on the chip surface. First, following a standard piranha cleaning bath, a metal multilayer consisting of 4 nm of chromium (used as adhesion layer) and 100 nm of gold is deposited over the entire chip surface using a magnetron sputtering system. Next, a thermal annealing process is performed, involving a temperature ramp of 10 °C/min up to 470 °C, a 60 min dwell at this temperature, and subsequent passive cooling. This treatment ensures the electrical resistivity reaches a stable value and prevents drifts that could compromise the stability of the phase-shifting operation.^18^ Then, the shifters are patterned by femtosecond laser ablation resulting in resistors with 8 um width and 3 mm length. In this way, heaters with a resistance value of 240 Ω can be fabricated. When a voltage is applied, Joule heating induces a localized temperature rise; the asymmetric placement of the heater relative to the two waveguides causes a difference in pressure. Via the thermo-optic effect (*dn*/*dT* ≈ 7 × 10^–6^ /K for borosilicate glass), this results in a tunable optical path length difference between the interferometer arms. Stability measurements on all heaters showed negligible variation (<0.2 Ω/h) of the value of the resistance during long operation at 12 V.

Each device includes two independent thermal phase shifters. The first is located within the MZI and allows tuning of the output power balance from a balanced configuration (SR = 50%) to a completely unbalanced one (SR = 100%), enabling full contrast modulation of the interference pattern. The second is positioned after the MZI and introduces a relative phase shift between the two output beams. This phase shift translates directly into lateral displacement of the structured pattern on the sample.

Using a thermal effect to achieve phase shifting makes the device prone, in theory, to exhibit thermal crosstalk between the two integrated resistors. In practice, however, we have neglected inter-resistors thermal crosstalk due to the geometry of the chip. In the current chip geometry (end-to-end distance between resistors of 4 mm, chip thickness 1 mm) the distance between the two thermal phase shifters is significantly larger than the distance between the resistors and the bottom surface of the chip. Having ensured good thermal contact between the bottom of the pattern generator and an aluminum heat sink, the thermal crosstalk between the two phase shifters can be safely assumed to be negligible. Furthermore, a previous study on a larger similarly designed device^19^ showed that in compatible conditions the crosstalk could be neglected.

In this configuration, both the amplitude and phase of the output pattern can be controlled using compact, monolithically integrated components. The design is scalable and modular, supporting multiwavelength operation and providing key functionality for SIM applications without moving parts or free-space optics.

### Mask Application for Background Removal

In integrated photonic systems, not all the input light couples efficiently into the waveguides. Uncoupled light can propagate through the transparent substrate and reach the output facet, resulting in a diffuse, noninterfering background that degrades the contrast of the generated illumination pattern. This background is particularly detrimental in structured illumination microscopy, where high pattern contrast is critical for achieving resolution enhancement and optical sectioning. To address this, we implemented a custom-fabricated output mask that blocks stray light while preserving the guided output beams, thereby enhancing the quality and fidelity of the structured illumination.

Light is coupled into the device by butt coupling a commercial polarization-maintaining optical fiber to the waveguide input and securing it to the chip’s facet with UV-curing glue (DELO PHOTOBOND GB345). Light that fails to be coupled into the waveguides - due to mode mismatch or misalignment – can propagate freely inside the device. Because the refractive index of the substrate is significantly higher than that of air, this uncoupled light will be guided along the device’s length and will not be able to exit through the top and bottom chip surfaces. Therefore, most of the uncoupled light will be able to reach the output facet of the device and will therefore propagate collinearly with the output of the chip, providing a significant background in illumination. To suppress this background, an external mask is fabricated and transferred to the output facet of the chip. The mask is fabricated by first sputtering a 200 nm thick layer of gold onto a glass substrate; afterward, a femtosecond laser is used to ablate two small (20 μm sides) holes at a distance equal to the outputs distance on the gold layer. The mask is aligned with the device outputs and the two are glued together using UV-curing glue. Afterward, the glass substrate on which the mask was fabricated is removed leaving only the gold mask attached to the chip. Removing the glass surface is essential to eliminate the double reflections that would be caused by the presence of a glass slab. As a result, an effective stray light rejection is achieved, eliminating almost completely the background illumination.

The degree of obtainable background rejection was estimated by measuring the total power at the output of the chip before and after mask application by placing a power meter at a close distance to the output facet of the chip. Afterward, the power coupled into the single waveguides is measured by imaging the chip facet at high magnification and selectively measuring the power exiting the waveguide by removing background light with a pinhole. In this way, it is possible to obtain an estimate of the optical power of the background light with respect to the one coupled into the waveguides. Measurements showed that the background power amounts to 34% of the total power at the chip’s output facet before mask application and to <1% after mask application.

The application of the external gold mask significantly reduces background illumination originating from uncoupled or scattered light within the substrate. By confining light transmission to two sharply defined apertures aligned with the output waveguides, the mask enhances the contrast of the interference pattern at the sample plane. This passive background suppression method is essential for maximizing signal-to-noise ratio in structured illumination imaging modes and contributes to the overall performance and robustness of the integrated illumination system.

### Phase Calibration

Structured illumination microscopy requires at least three pattern translations to separate frequency bands and achieve higher-resolution reconstructions, which in our device are implemented via thermo-optic phase shifters imposing a controllable phase difference between the light traveling in two different waveguides through voltage-dependent Joule heating. Characterizing the relationship between applied voltage across the heaters and resulting phase shifting can be performed by producing structured illumination onto a fluorescent sample and observing the resulting fluorescence image while varying the voltage applied across the shifters. For estimating the phase shift induced by the phase shifter of the device, assuming zero offset phase for the image with no voltage applied, the phase shift is estimated with the method presented in,^20^ which consists of computing the Fourier transform of the image, finding the peaks corresponding to the pattern frequency and extracting the pattern translation as the complex phase of the peak.

Calibration of the first thermal shifter of the device, which is used to perform amplitude modulation, can be performed more straightforwardly by finding the voltages at which the pattern contrast is maximized and minimized, corresponding respectively to the configurations in which power is evenly split between the outputs and in which light is present in only one waveguide.

## Results and Discussion

### Phase Control and Stability

Precise and stable phase control is essential in structured illumination microscopy, where consistent and reproducible fringe positioning directly affects image reconstruction quality. In particular, maintaining phase stability across time is crucial for quantitative imaging and multiframe acquisitions, while ensuring repeatability across different days enables robust system calibration and reproducible experimental protocols. If no phase retrieval algorithm is applied to the raw SIM data, phase inaccuracies larger than 2% can result in artifacts in the reconstruction process. However, a posteriori algorithms can be used successfully to deduce the pattern translations directly from the acquired images, enabling artifact-free SIM reconstructions to be obtained even with nonideal phases.^20,21^

To assess the stability and repeatability of the phase shifter, we performed repeated measurements over time of the phase-voltage characteristic curve of the second phase shifters. Stability was evaluated by acquiring data every 45 min within a single day (Figure 2a), while repeatability was assessed by performing the same experiment daily over the course of a week (Figure 2b). All these measurements were performed by keeping the device turned off in between measurements. The standard deviation relative to the average phase value was 1.8% for stability and 5.5% for repeatability. These results demonstrate that the thermo-optic phase shifter maintains good stability over short time scales, supporting multiframe acquisitions without the need for repeated calibrations. However, increased variability observed over longer durations is likely attributable to fluctuations in the laboratory environment, where temperature is not controlled. To account for this limitation the system must undergo periodic recalibration, or phase retrieval algorithms are required for accurate SIM image reconstruction. To ensure low phase error during SIM imaging, specifically, a daily calibration was performed before each measurement session, and a reconstruction algorithm including an a posteriori phase retrieval step^22,23^ was employed.

**Figure 2:**
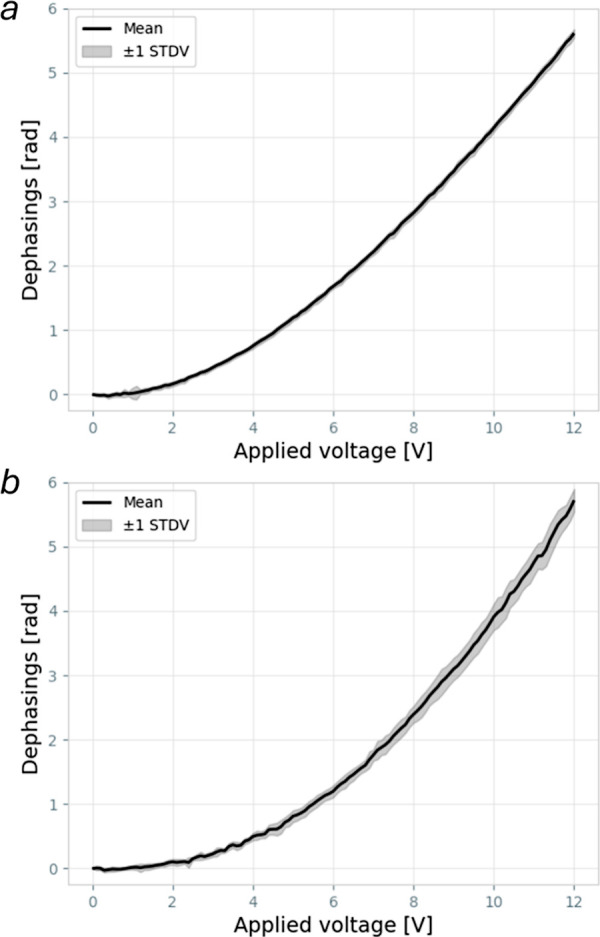
Phase shifting characteristic curve measurements, obtained performing multiple phase shifting measurements across a single day (a) and once a day for 7 days (b).

### Contrast Measurement

One of the key parameters for assessing the quality of a generated pattern is its contrast, defined as the ratio between the pattern amplitude and its average intensity. In both HiLo microscopy and SIM the contrast of the interference pattern is directly proportional to the achievable signal-to-noise ratio (SNR). As such, pattern contrast serves as a critical figure of merit for evaluating the performance of our device. However, the contrast of the interference pattern cannot be reliably measured by imaging the fluorescence emission from a uniformly labeled sample. This is because the microscope’s finite spatial resolution, described by its modulation transfer function (MTF), attenuates high spatial frequencies, leading to an underestimation of the true pattern contrast, particularly for fine interference fringes. To overcome this issue, we imaged a sparse distribution of subdiffractive fluorescent beads (PS-Speck P-7220), which serve as near-point sources and sample the local excitation intensity without significant spatial averaging. The pattern contrast was measured by equalizing the power of the two output beams via the MZI phase shifter and by translating the illumination pattern using the integrated thermal phase shifter (Figure 3), resulting in a measurement of the fluorescent intensity of each bead as a function of the phase shift applied to the pattern. For each bead, the maximum (*M*) and minimum (*m*) fluorescence intensities were extracted, and the contrast was computed as *C* = (*M* – *m*)/(*M* + *m* – 2*b*), where *b* represents the camera dark noise level. From the analysis of the 100 brightest beads in the field of view, we obtained an average pattern contrast of 91%, with a standard deviation of 4.98%. This high contrast confirms the capability of the device to produce well-defined interference patterns suitable for high-SNR imaging applications. Residual variability likely stems from local phase nonuniformities or bead-specific factors such as depth within the sample or signal saturation.

**Figure 3:**
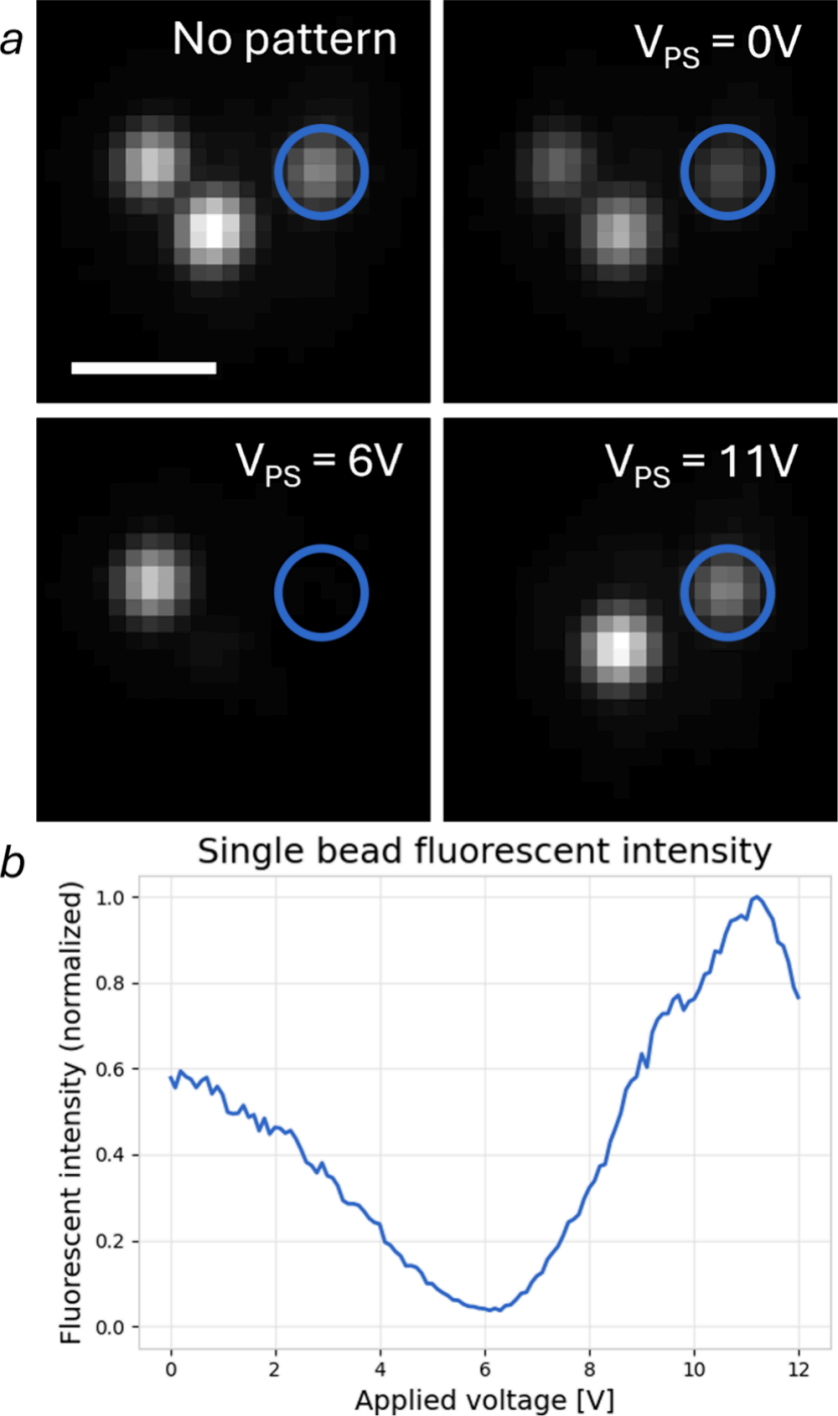
Contrast measurement with nanobeads sample. (a) Images of three fluorescent beads illuminated with uniform (top left) and structured illumination while applying a voltage of 0, 6, and 11 V to the integrated resistive thermal shifter to translate the pattern. Scalebar is 1 μm. (b) Normalized fluorescent intensity as a function of applied voltage.

### Intensity Balance at Multiple Wavelengths

Typically, directional couplers are used in integrated optics to split light between two waveguides with a fixed power ratio. However, the SR of such couplers depends on the evanescent modes overlap between adjacent waveguides and, thus, it is strongly wavelength dependent. As a result, when a single directional coupler is used across multiple wavelengths, the power distribution between the output arms varies with wavelength—leading to a wavelength-dependent contrast in the generated interference pattern. To address this limitation, we adopted an MZI configuration, which allows active tuning of the output power balance over a broad spectral range. An important analytical result in this regard is that an MZI incorporating two unbalanced beam splitters can still achieve equal output powers (i.e., a balanced configuration) provided that the SR of each beam splitter lies between 14.6 and 85.4%. This result can be obtained by considering an MZI composed of two identical, unbalanced directional couplers with splitting ratio R and a phase shifting element inducing a phase difference φ between the two waveguides. Computing the transfer matrix of such a device, it can be derived that the ratio between the output powers of the two waveguides is equal to 1 – 4*R*(1 – *R*)­cos^2^(φ/2).^24,25^ Therefore, by tuning the induced phase shift the device can assume any value of SR between 1 – 4*R*(1 – *R*) and 1. It is worth noting that any arbitrarily unbalanced Mach–Zehnder configuration is able to reach an SR of 1, meaning that uniform illumination can always be obtained using a device with a MZI configuration by illuminating the sample with a single beam. To obtain structured illumination with optimal interference contrast, instead, an SR of 1/2 is needed; therefore, the minimum SR obtainable from the unbalanced MZI must be larger than 1/2, i.e., One – 4*R*(1 – *R*) ≤ 1/2. Solving this inequality, it can be proven that the SR of the single directional couplers must be in the range [0.25(2 – √2), 0.25­(2 + √2)], which corresponds to the target 14.6–85.4% range.

The directional couplers fabricated in the devices were optimized for a working wavelength of 638 nm, obtaining a SR of 49.1%, and showed a SR for 561 nm working wavelength equal to 81.9%. Therefore, the device is theoretically able to obtain a balanced configuration at both 561 and 638 nm working wavelengths. This capability was verified experimentally by adjusting the relative phase shift between the two arms of the interferometer and measuring the SR at both wavelengths. In this way, we could achieve balanced output powers at both 638 and 561 nm (Figure 4). This wavelength-independent balance enables high-contrast multicolor structured illumination. An important limitation that must be mentioned is that both thermal shifters (for amplitude and dephasing control) rely on induced phase differences Δφ in the optical circuit depending on the wavelength as follows:

$$\Delta \varphi (V)=\frac{2\pi }{\lambda }\Delta n(\Delta T(V))L$$

where *V* is the actuation voltage, *L* is the length of the shifter, Δ*T* is the local temperature of the waveguide with respect to the substrate, *n* is the refractive index and λ is the wavelength. This means that this device is unable to perform simultaneous balancing of multiple colors, since the voltage that needs to be applied to the MZI thermal phase shifter in order to obtain a 50% splitting ratio varies with wavelength. Second, given the explicit wavelength dependence of the resulting dephasing, applying a voltage to the second thermal phase shifter would cause different phase translations of the interference pattern generated at the pattern plane, meaning that the same voltage cannot be used for multiple wavelengths if the same dephasing is desired. Both of these issues make simultaneous multicolor SIM acquisition unfeasible; therefore, multicolor acquisitions have been performed sequentially.

**Figure 4:**
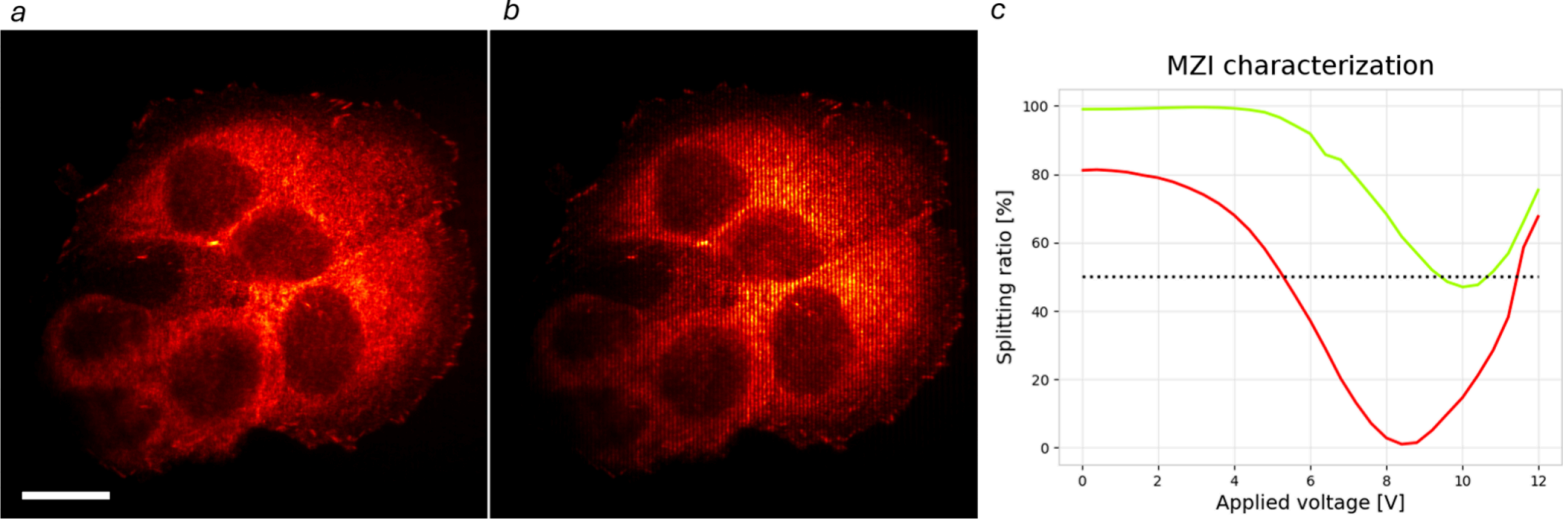
Mach–Zehnder interferometer characterization and multicolor behavior. (a,b) Fluorescent images of MCF7 cells agglomerate with AF647 vinculin, observed with uniform (a) and structured (b) illumination at 638 nm excitation. Scalebar is 20 μm. (c) Characterization of the splitting ratio as a function of the voltage applied to the Mach–Zehnder interferometer thermal phase shifter for 638 and 561 nm wavelengths. The black dotted line marks a splitting ratio of 50%, i.e., balanced power splitting between the two chip outputs.

### Optical Sectioning with High and Low Frequency Hybrid Illumination (HiLo)

One application of patterned light in microscopy is an optical sectioning technique designed to enhance image contrast and resolution in wide-field fluorescence microscopy by effectively reducing out-of-focus light, called HiLo microscopy.^2,26^ The method involves capturing two images of the specimen: one under uniform illumination and another under structured illumination, such as laser speckle or fringe patterns. The uniform illumination image contains both in-focus and out-of-focus information, while the structured illumination image emphasizes in-focus details due to the high contrast of the imposed pattern, which diminishes for out-of-focus regions. By computationally combining the high-frequency components from the uniform illumination image with the low-frequency components from the structured illumination image, HiLo microscopy reconstructs a high-contrast, optically sectioned image that effectively suppresses background haze. This approach is particularly advantageous for real-time, in vivo imaging applications, offering a simpler and faster alternative to confocal microscopy without the need for point scanning.

The ability to perform HiLo microscopy with the proposed device was tested by imaging a fixed slide containing a 16 μm-thick mouse kidney section where F-actin was stained with phalloidin conjugated to Alexa Fluor 568 (Invitrogen FluoCells prepared slice #3). An illumination wavelength of 561 nm was used to optimally excite the fluorophore. The microscope objective lens used was a 60×, 1.49 NA oil immersion objective (Nikon CFI Apochromat TIRF 60XC Oil). After acquiring images with both uniform and structured illumination for each z section of the sample, HiLo reconstruction was computed using the algorithm described in^27^ the difference between the two images of the sample is computed and its absolute value is obtained, providing an estimate of the regions in which the pattern is visible in the structured image. Afterward, the uniform image and the difference image are high-pass and low-pass filtered, respectively, using two complementary Gaussian filters with the same threshold frequency, which was equal to half the spatial frequency of the structured pattern. Lastly, the two images are stitched together in Fourier domain, recovering an image of the in-focus signal without incurring in loss of lateral resolution. In the resulting image (Figure 5), different sections of the proximal convoluted tubules and the brush border of the kidney sample can be clearly distinguished and a noticeable out-of-focus light rejection is observed, proving the efficacy of this approach to perform HiLo microscopy.

**Figure 5:**
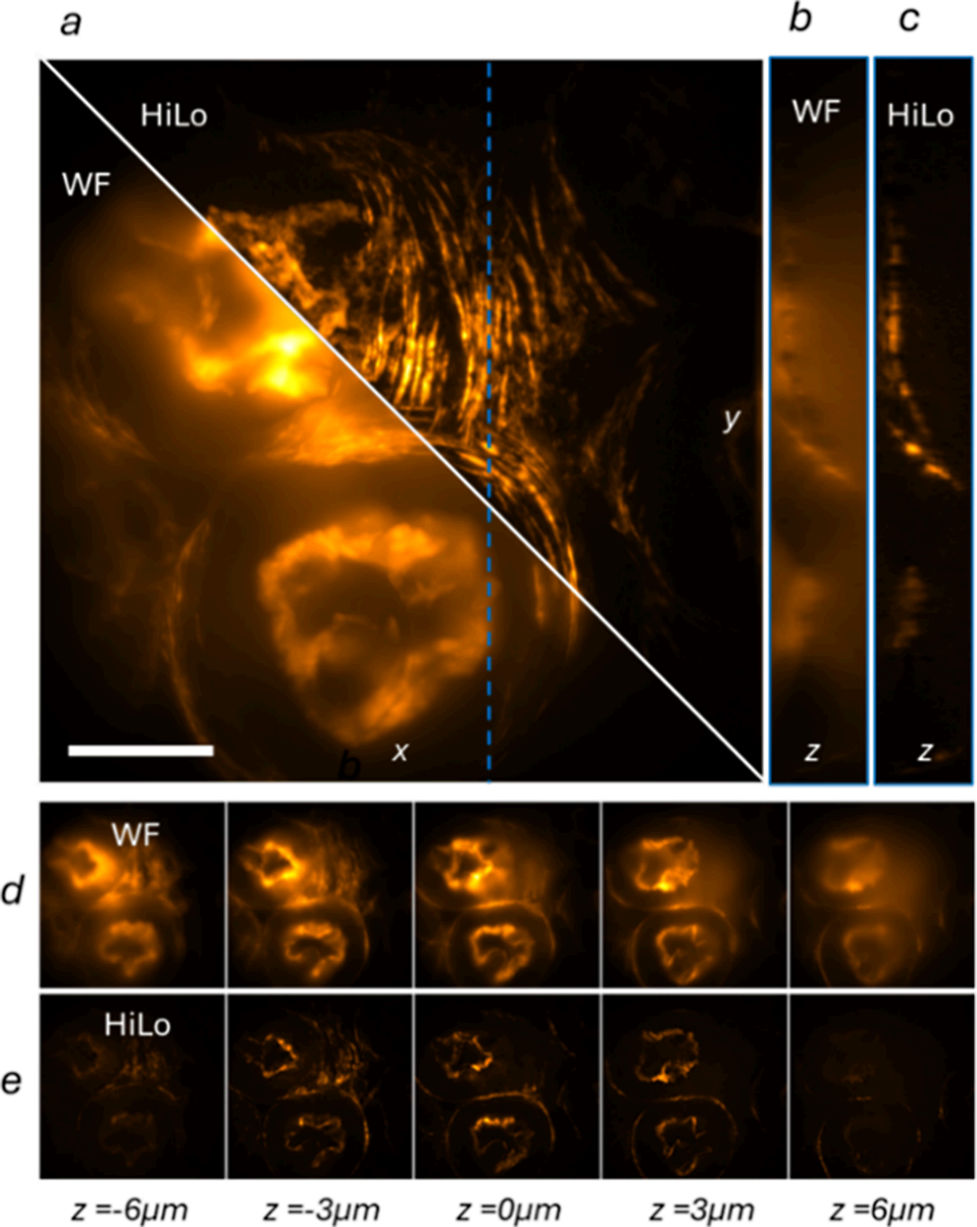
HiLo imaging of kidney slices. (a) Maximum Intensity Projection (MIP) of a mouse kidney section (Fluocells prepared slide #3, Invitrogen) observed at 561 nm excitation using standard widefield (WF) microscopy and HiLo. Scalebar is 20 μm. (b) Cross-sectional view of a single plane obtained with WF microscopy, corresponding to the vertical blue dotted line in (a). (c) Cross-sectional view of the corresponding plane reconstructed using HiLo. (d) Multiple optical sections acquired in WF mode at 3 μm steps. (e) Corresponding optical sections reconstructed with HiLo.

The HiLo reconstructions obtained with the integrated photonic pattern generator demonstrate effective optical sectioning and background suppression in a thick biological specimen. Compared to conventional widefield imaging, the HiLo images exhibit a reduction of out-of-focus fluorescence, resulting in improved contrast and enhanced visibility of fine structural features such as the brush border and proximal convoluted tubules. Importantly, the preservation of lateral resolution in the reconstructed images confirms that the computational fusion of uniform and structured illumination components does not introduce spatial blurring. These results validate the suitability of the proposed integrated device for HiLo microscopy and highlight its ability to deliver optical sectioning performance comparable to established implementations, while benefiting from the intrinsic stability, compactness, and programmability of the on-chip illumination platform.

### Multicolor Structured Illumination Microscopy

Structured Illumination Microscopy (SIM) is an advanced imaging technique that enhances the resolution of a widefield microscope beyond the diffraction limit by using patterned illumination.^3,4,28^ In SIM, a high-frequency periodic light pattern (typically stripes) is projected onto the sample and shifted or rotated in multiple orientations. When this structured light interacts with subdiffraction features in the sample, it generates Moiré fringes that encode high-resolution spatial information. By computationally reconstructing the final image from multiple shifted patterns, SIM effectively doubles the lateral resolution of conventional widefield microscopy, reaching resolutions down to ∼100 nm. Exploiting the MZI configuration of the device, both 561 and 638 nm illumination wavelengths were employed to perform SIM (Figure 6); as previously mentioned, images at the two wavelengths were acquired sequentially. The sample consisted of fixed MCF7 cells stained with TRTC-phalloidin to visualize F-actin, while vinculin at focal adhesion sites, appearing as point-like subdiffraction structures, was labeled with Alexa Fluor AF647. This sample was selected to simultaneously assess SIM performance on both point-like and extended biological structures. Vinculin localized at focal adhesion sites forms subdiffraction, quasi-point emitters, making it well suited for evaluating the effective point spread function and quantitatively estimating lateral resolution enhancement. In contrast, the filamentous F-actin network provides complex yet well-defined extended structures, enabling qualitative assessment of contrast improvement, background rejection, and structural fidelity in reconstructed images.

**Figure 6:**
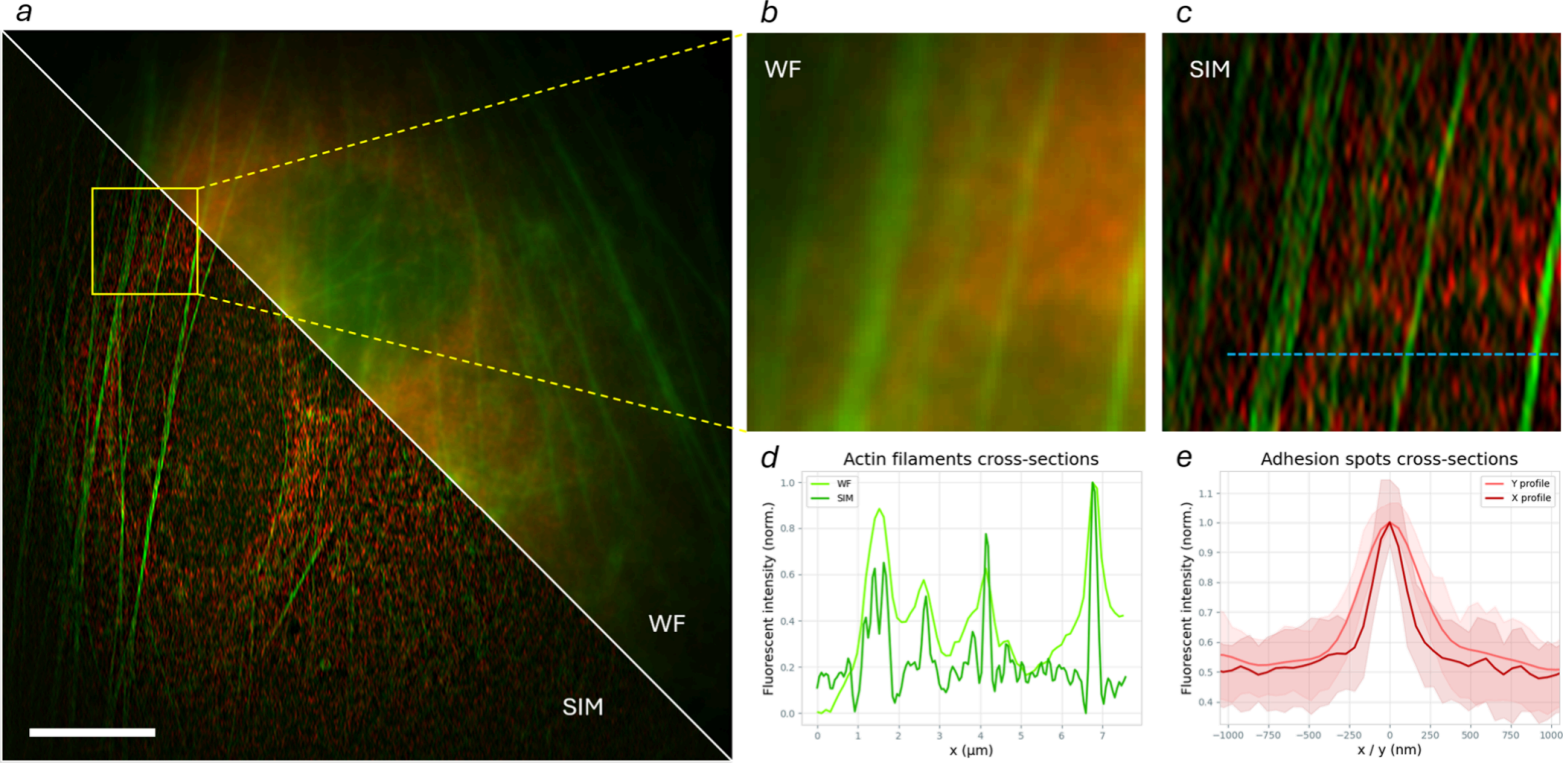
Dual-color 1D-SIM imaging. (a) Dual-color SIM experimental results of fluorescence imaging of MCF7 cells with phalloidin and vinculin staining observed using excitation light at 561 nm (green channel) and 638 nm (red channel), showing both the widefield image (top right) and the reconstructed SIM image (bottom left). Scalebar is 20 μm. (b,c) Close-up of a detail of image in widefield (b) and SIM (c) modalities. (d) Line plot along the blue dashed line of the green channel, showing a cross-sectional view of the actin fibers. (e) Comparison between the average vertical and horizontal cross sections of the vinculin-labeled focal adhesion sites, highlighting the resolution enhancement along the horizontal direction. The shaded areas represent the first and third quartiles of the normalized distributions.

After image acquisition and reconstruction, resolution enhancement and background rejection can be observed in both the green and red channels. Since the integrated chip generates two horizontally aligned beams, the resulting structured illumination pattern is oriented along the *x*-axis: consequently, resolution enhancement is achieved only along the horizontal image direction.

To assess the resolution improvement, the ratio between horizontal and vertical width of the images of the vinculin focal spots was measured (Figure 6e) and resulted in an average resolution enhancement of 1.71 along the horizontal direction, closely matching the theoretically computed value of 1.75. A limitation of this pattern generation scheme is the inability to change the generated pattern’s spatial frequency and, consequently, the achievable resolution enhancement. These values depend on the distance between the outputs of the pattern generator and the optical magnification between them and the back focal plane of the objective lens, and neither of these parameters can be changed straightforwardly.

The measured lateral resolution enhancement factor closely matches the theoretical value expected for linear SIM given the illumination spatial frequency and objective numerical aperture. This result demonstrates that the integrated photonic device reproduces the performance of conventional one-dimensional SIM under equivalent conditions, while simplifying the illumination hardware and improving its mechanical robustness. Overall, this agreement between theoretical expectations and experimental measurements confirms that the proposed integrated photonic approach delivers quantitatively reliable SIM performance while offering a compact, stable, and alignment-insensitive alternative to conventional free-space implementations.

## Conclusions

We have presented a novel integrated photonic device for structured illumination, offering precise amplitude and phase control. By integrating a metal mask at the output facet, the device significantly enhances contrast and suppresses background noise, addressing key limitations observed in previous implementations. These improvements enable high-contrast sinusoidal illumination patterns, facilitating the integration into conventional widefield microscopes.

Our results demonstrate the potential of this device for HiLo microscopy and multicolor SIM, highlighting its effectiveness in optical sectioning and resolution enhancement. Although the primary objective of this work is the development of a versatile photonic illumination platform, the demonstrated HiLo optical sectioning in thick tissue and dual-color SIM imaging in cells illustrate direct relevance to biological imaging. In particular, the ability to switch between uniform, HiLo, and SIM illumination using the same device could be advantageous for low-phototoxicity and live-cell imaging.

In its current implementation, the sinusoidal pattern is generated along a single axis due to the use of a chip with two waveguides. While this scheme is already attractive for optical sectioning and potentially for single-molecule localization with improved axial resolution,^29^ future developments will explore the integration of multiple waveguides in triangular or hexagonal configurations, further expanding the capabilities of integrated photonic devices for 2D and 3D structured illumination microscopy.^30^ By leveraging the three-dimensional flexibility of our waveguide fabrication process (femtosecond laser micromachining), these advancements could enable more complex illumination patterns for further improvements in spatial resolution.

The proposed integrated photonic chip represents a significant step toward compact, prealigned structured illumination microscopy. By combining precise optical control of the light pattern without moving parts, this technology has the potential to advance imaging and photonic integration, providing a robust platform for optical microscopy and beyond.

## Data Availability

All data needed to evaluate the conclusions in the paper are present in the paper. The raw data for the images presented in the manuscript are available as an open data set on Zenodo (doi: 10.5281/zenodo.17046940). The code to reconstruct the SIM data is available as a Napari plugin at https://napari-hub.org/plugins/napari-sim-processor.html.

## Abbreviations

DC: directional coupler
HiLo: high- and low-frequency illumination
MTF: modulation transfer function
MZI: Mach–Zehnder interferometer
SIM: structured illumination microscopy
SNR: signal-to-noise ratio
SR: splitting ratio
